# Diagnosis and Classification of Acute Leukemia in Bone Marrow Trephine Biopsies, Utility of a Selected Panel of Minimal Immunohistochemical Markers

**Published:** 2016-07-01

**Authors:** Priya Subashchandrabose, Lakshmikanth Ramiah Madanagopaal, Tadury Madhukar Subba Rao

**Affiliations:** 1 Assistant Professor, Department of Pathology, Saveetha Medical College, Chennai, India; 2 Chief of Lab and Consultant Pathologist, Department of Pathology, Dr Lal Path Labs, Chennai, India; 3 Professor, Department of Pathology, PSG Institute of Medical Sciences and Research, Coimbatore, India

**Keywords:** Acute leukemia, Classification, Diagnosis, Immunohistochemistry, Trephine biopsy

## Abstract

**Background:** Acute leukemias are characterized by neoplastic proliferation of hematopoietic stem cells and accumulation of blasts and immature cells in bone marrow. We applied a selective panel of immunohistochemical markers on bone marrow trephine tissue sections and observed their utility in diagnosis and typing of acute leukemia.

**Materials and Methods:** The study was done at PSG Institute of Medical Sciences and Research from 1st January, 2008 to 30th June, 2012. Immunohistochemistry was done to detect the expression of Myeloperoxidase (MPO), Terminal deoxynucleotidyl transferase (TdT), Cluster of Differentiation 3 (CD3) and Cluster of Differentiation 20 (CD20).

**Results:** On an average, 76 new cases of leukemia are diagnosed each year in our hospital. Of these 28.7% are acute leukemias, which had a bimodal peak age of occurrence with almost equal sex distribution. Only 9 cases could be typed as Acute Myeloid Leukemia (AML) or Acute Lymphoid Leukemia (ALL) purely by morphology. Another 10 cases were typed using cytochemistry. Immunohistochemical panel helped to type 90% of cases. We also identified 1 case of AML of ambiguous lineage. The data were analysed statistically using SPSS version 21 and found out that the immunohistochemistry was found to be extremely significant (p<001) by Chi-Square test.

**Conclusions:** Based on our results, we suggest the use of this limited panel of markers for routine evaluation of all acute leukemias. It is easier to type using immunohistochemistry rather than flow cytometry, given the disadvantage of the costs involved with the latter.

## Introduction

 Acute leukemias are characterized by neoplastic proliferation of hematopoietic stem cells and are broadly classified into two main groups namely: Acute Myeloid Leukemia (AML) and Acute Lymphoid Leukemia (ALL) based on the cellular presentation of primary stem cell defect.^1^ World Health Organization (WHO) further subclassifies them based on morphology, cytochemistry, immunophenotyping, cytogenetic and molecular genetics studies.^2^ In our institute, the diagnosis and typing of acute leukemias rested principally on morphological assessment and cytochemical studies. In few cases, the typing could be established but was not possible in some cases. We, therefore, applied a minimal panel immunohistochemical marker on the bone marrow trephine tissue sections and observed their utility in diagnosis and typing of acute leukemia.

## MATERIALS AND METHODS

 The study was done at PSG Institute of Medical Sciences and Research, Coimbatore. The study period was 4.5 years from 1st January, 2008 to 30th June, 2012. The peripheral smear registers were accessed, cases diagnosed as acute leukemia were noted and by logging into our hospital information system, the bone marrow aspiration and trephine biopsy of these cases were noted. We reviewed all the slides with reports of results and particular attention to the morphology of the blasts. The cytochemistry slides wherever available were also reviewed.

Finally, we shortlisted cases which had all three diagnostic material needed to complete our study, namely, peripheral smear, bone marrow aspiration smear and bone marrow trephine biopsy specimen and blocks. Paraffin blocks of the trephine biopsies of the study population and following normal tissues (spleen for MPO, thymus for TdT and tonsils for both CD3 and CD20) to serve as controls were chosen and fresh sections of 4 µm thickness were cut. Slides were deparaffinised, rehydrated, treated with 3% Lugol’s iodine and cleared with 2.5% sodium thio sulphate. Antigen retrieval was done using a pressure cooker.

Primary antibodies available in liquid form including Anti–CD3 (T cell) mouse monoclonal [clone, PS1], Anti–CD20 (B cell) mouse monoclonal [clone, L-26], Anti–Myeloperoxidase (MPO) rabbit polyclonal and Anti–Terminal deoxynucleotidyl transferase (TdT) mouse monoclonal [clone, TdT 88] (Biogenex, San Ramon, USA) were used.

The incubation time for MPO, CD20, CD3 and TdT was 30, 30, 60 and 120 minutes at room temperature, respectively as recommended by manufacturer (Biogenex). Evaluation of IHC staining was performed, observations were documented and results were analyzed.

## Results

**Figure 1:**
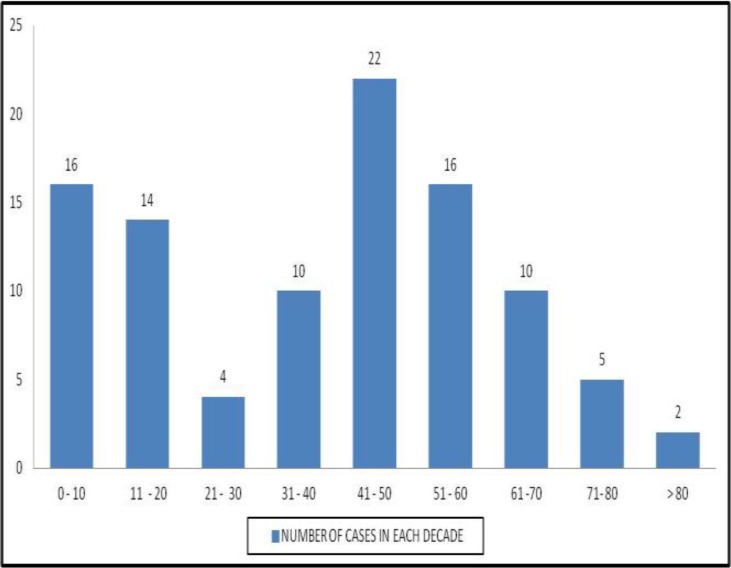
Age distribution

 During the study period, out of total 344 cases reported as leukemia, 99 cases were diagnosed as acute leukemia comprising 28.7% of the total leukemias. There was a bimodal peak age of occurrence i.e. 0-20 years and 41-60 years. The mean age was 39 years. Age distribution of the cases of acute leukemia is shown in Figure 1.

**Figure 2:**
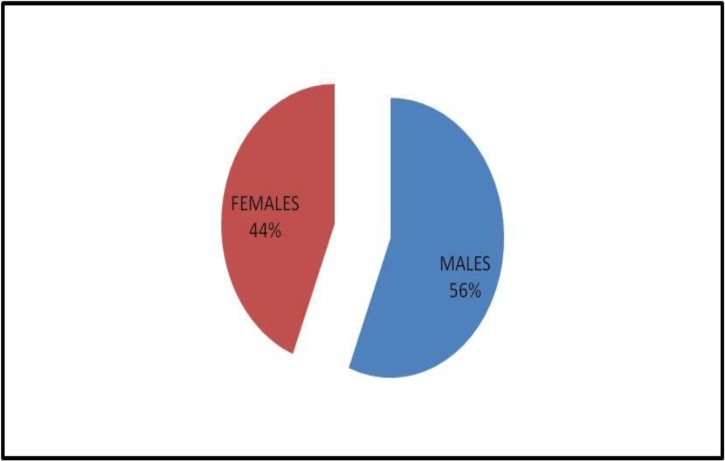
Sex distribution

The sex distribution of cases of acute leukemia is shown in Figure 2.

**Figure 3:**
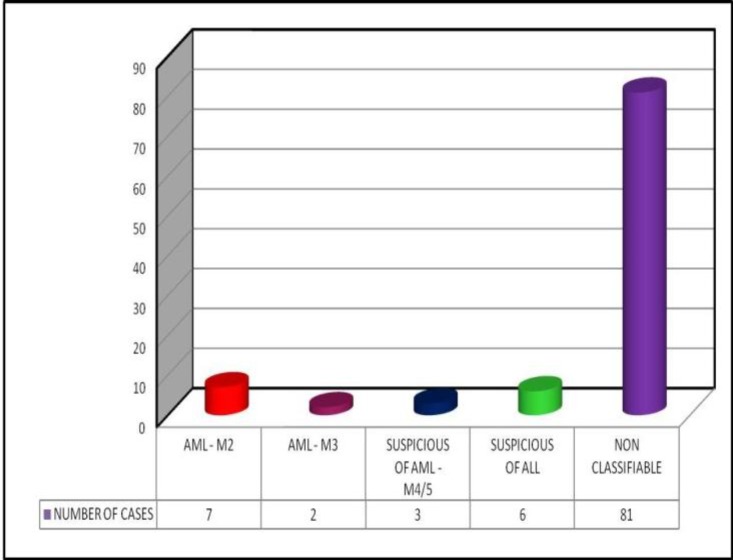
Distribution of types of acute leukemia reported based on morphology alone

Males slightly outnumber females. Of the 99 cases diagnosed as acute leukemia by the peripheral smear/bone marrow aspirate/both, a definitive diagnosis of the type of acute leukemia based purely on the morphology was possible only in 9 cases (AML–M2 seven cases and AML–M3 two cases) as shown below in Figure 3.

**Figure 4:**
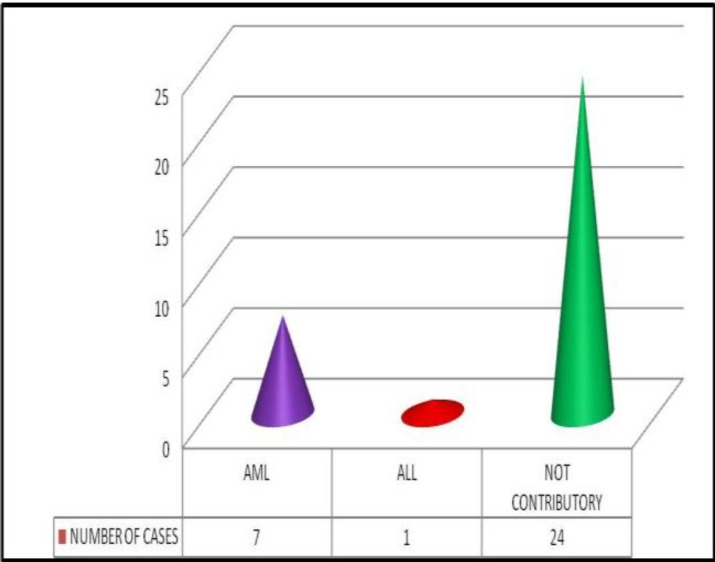
Distribution of acute leukemia types following cytochemical studies

Cytochemistry was done only in 32 of the cases. Of these, a definitive diagnosis on the type of leukemia was made on 8 cases (25%) shown in Figure 4.

7 cases were AML (myeloperoxidase positive) and 1 was diagnosed as ALL (block positivity with periodic acid Schiff).

**Figure 5:**
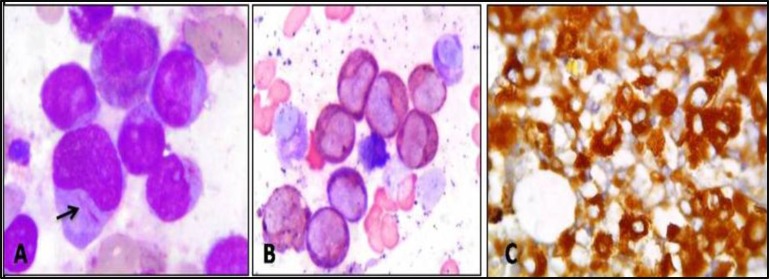
Case of AML M2. A: Peripheral smear reported as AML-M2 with an Auer rod in a myeloblast (arrow).

A case of AML–M2 diagnosed by morphology on the peripheral smear, the cytochemistry was corroborative by showing myeloperoxidase positivity and immunochemistry also confirmed the positivity for myeloperoxidase (Figure 5).

**Figure 6:**
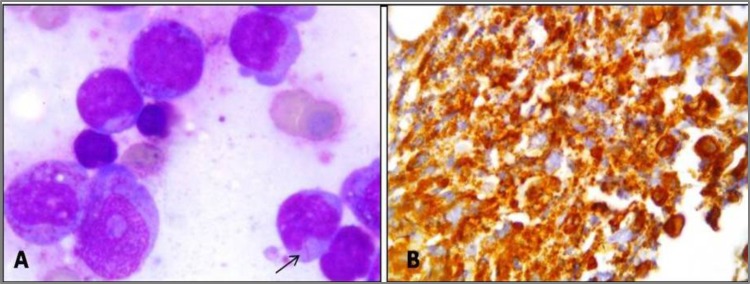
Case of Acute promyelocytic leukemia. A: Bone marrow smear reported as AML-M3 showing many promyelocytics and a myeloblast with an Auer rod (arrow); Giemsa; x1000. B: Myeloblasts staining intensely positive for myeloperoxidase on IHC study; x1000.

Another case diagnosed by morphology on bone marrow aspirate as AML–M3 and the immunochemistry was also confirmatory by showing intense myeloperoxidase positivity in the myeloblasts (Figure 6). The summary of the results of utility of Cytochemistry, for the cases diagnosed as acute leukemia, AML, ALL and suspicious of AML M4/M5 by morphology in the peripheral smear and bone marrow aspirate is shown in Table 1.

**Figure 7:**
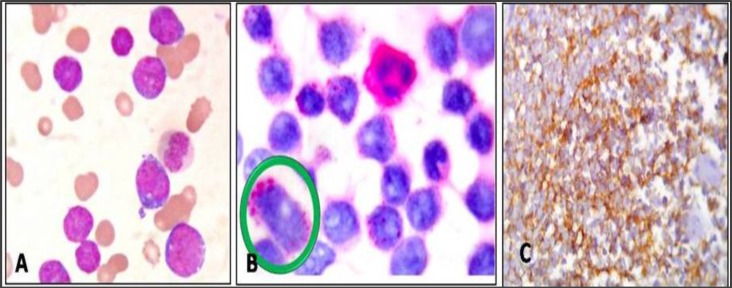
Case OF ALL. A: Peripheral smear reported as ALL-L2. Blast showing variation in size, occasional nucleoli and variable amounts of cytoplasm; Leishman; x400. B: Blast typical block positivity with PAS stain. The cytoplasm of the neutrophil which stains diffusely positive, serves as an internal control; x400. (Insert circle shows lymphoblast with large magenta colored block; PAS; x1000). C: Lymphoblasts showing positivity for CD20 ON IHC study; x400.

A case of ALL diagnosed by cytochemistry (block positivity in the cytoplasm) and immunohistochemistry showed positivity for CD20, thus confirming the diagnosis (Figure 7).

Out of the total study population of 36 acute leukaemias, only 16 cases were diagnosed as AML and ALL by morphology and cytochemistry. The Table 2 shows the summary of the diagnosis of these 36 cases before the application of immunohistochemistry.

**Table 1:** Summary of cytochemistry results

**Morphologic ** **diagnosis on ** **PS/BMA smears**	**Cytochemistry**		**Utility of results of ** **cytochemistry (MPO ** **and PAS)**
	Done	Not done	
Acute leukemia	32	49	7 diagnosed as AML 1 diagnosed as ALL 24 not contributory
AML	7	2	6 corroborative 1 not contributory
Suspicious of ALL	6	0	3 diagnosed as ALL 3 not contributory
Suspicious of AML -M4/M5	3	0	Not contributory in all 3
Total	48	51	11 cases typed as AML or ALL 6 corroborative with morphological diagnosis 31 not contributory
Grand total	99		

**Table 2:** Diagnosis prior to the application of IHC studies

**Diagnosis**		**No of cases**	
Acute Leukemia		20	
AML	Diagnosis by morphology	6	11
	Diagnosis only after cytochemistry	5	
ALL	Diagnosis by morphology	0	5
	Diagnosis only after cytochemistry	5	
Total cases (study population for IHC studies)		36	

Leishman; x1000. B: Myeloblasts showing cytoplasmic granular positivity for myeloperoxidase; x400. C: myeloblasts staining intensely positive for myeloperoxidase on IHC study; x1000.

Of the 20 cases of acute leukemias, the IHC panel could type 18 of whom as either myeloid or lymphoid. Table 3 shows the subtypes of acute leukemia after the application of immunohistochemistry. Thus, IHC has been used to subtype 90% of patients with acute leukemia. 1 case did not express any of the phenotypes studied. Another case showed positivity for both lineages.

**Figure 8:**
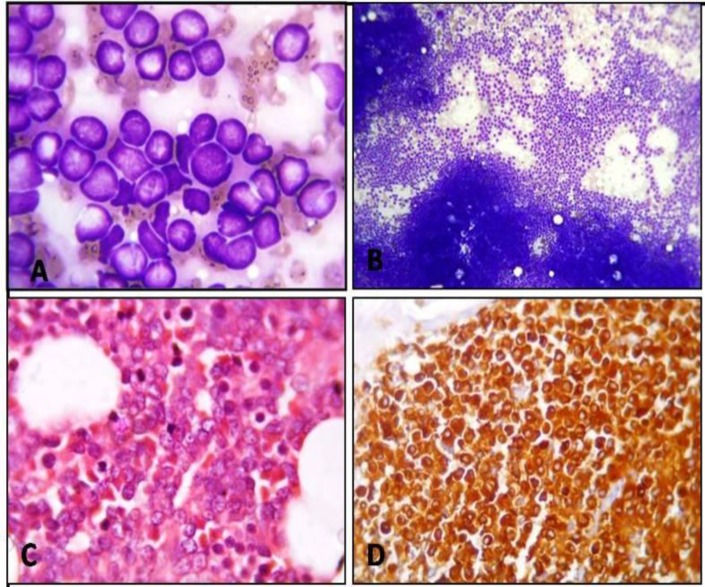
Case of AML. A: Peripheral smear reported as acute leukemia. Leishman; x1000. B: Hypercellular bone marrow aspirate smear composed of a monotonous population of undifferentiated blasts. Gimasa; x100. C: bone marrow triphine biobsy showing hypercellular marrow spaces replaced by undifferentiated blasts; H & E x400. D: Blasts are diffusely positive for myeloperoxidase on IHC; x400.

A case of acute leukemia which could not be typed by morphology and cytochemistry, it was diagnosed as AML only after the application of Immunohistochemistry (Figure 8).

The results of phenotyping by immunohistochemical studies on the cases of acute leukemia which could already be subtyped as AML and ALL based on morphology and cytochemistry is shown in the Table 4.

**Figure 9:**
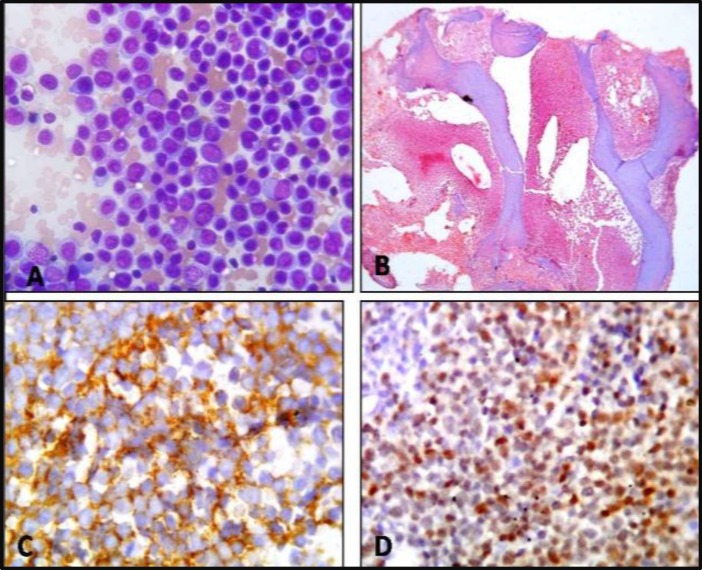
Case of ALL. A: Bone marrow reported as acute luekemia.Giemsa; x400. B: Bone marrow section showing extensive replacementof marrow spaces by blasts; H & E; X40. C: Blasts showing positivity for CD 20 by IHC; x400. D: Blasts are also positive for TdT by IHC x100.

Table 5 shows the overall summary of the phenotypes of the immature cells after the performance of IHC studies. 83% of ALL cases were of B cell lineage (CD20 positive) and 90% of them were TdT positive. 17% of ALL cases were of T cell lineage (CD3 positive) and 1 of them was TdT positive. None of the AML blasts were positive for TdT. Overall, 31% of ALL cases were positive for TdT. A case of acute leukemia which could not be typed by morphology and cytochemistry, it was diagnosed only after Immunohistochemistry as a case of ALL is shown (Figure 9).

**Table 3:** Subtypes of Acute Leukemia after IHC studies

**Subtype of acute leukemia after IHC study**	**No. of cases**
AML	11
ALL	7
Acute leukemia of ambiguous lineage	1
Not contributory	1
Total	20

**Table 4:** Results of IHC on acute leukemia already typed by morphology or cytochemistry

**Diagnosis**	**No of ** **cases**	**MPO**	**CD20**	**CD3**	**TdT**
AML	11	11/11	0/11	0/11	0/11
ALL	5	0/5	4/5	1/5	3/5

**Statistical analysis**

The data were analyzed statistically using SPSS version 21 by Chi-square test with Yates correction.

Table 6 shows the comparison of number of cases diagnosed by morphology and cytochemistry versus number of cases diagnosed by Immunohistochemistry. The Chi-square test was equal to 21.782 with 1 degree of freedom IHC is extremely beneficial in diagnosing leukemia when compared to diagnosing leukemia with aid of morphology and cytochemistry alone and it is statistically significant (p<0001).

## Discussion

 All acute leukemias are required to be classified either as AML or ALL. This is crucial for two interdependent reasons: one, to choose the most appropriate ancillary investigation for exact subtyping and second to offer the most appropriate therapy. The advent of targeted gene therapy has made it imperative for this subtyping to be done.^3^

In 1976, French-American-British (FAB) co-operative group classified AML into 6 sub groups (M1–M6) and ALL into 3 sub groups (L1–L3).^4^ In 1985, subtype named M7 was added to the FAB classification of AML.^5^

**Table 5:** Summary of the phenotypes of acute leukemia

**Diagnosis**	**No of ** **cases**	**MPO**	**CD20**	**CD3**	**TdT**
AML	22	22/22	0/22	0/22	0/22
ALL	12	0/12	10/12	2/12	10/12
Acute leukemia of ambiguous lineage	1	1/1	0/1	1/1	1/1
Not contributory	1	0/1	0/1	0/1	0/1
All leukemias	36	23/36	10/36	3/36	11/36

**Table 6:** Comparison of morphology and cytochemistry with IHC

**Diagnosis by**	**Positive**	**Negative**	**Total**
Morphology and cytochemistry	16	20	36
Immunohistochemistry	35	1	36
Total	51	21	72

In addition to the morphology, immunophenotyping was also used to diagnose this subtype.^6^ In 1991, M0 was included in the classification of AML. Immunophenotyping and electron microscopy were used to differentiate this subtype as the morphology of AML M0 and ALL L2 blasts were very similar.^7^ In 1999, the WHO and the International Society of Hematology proposed a new classification of acute leukemias which was published in 2001. In 2004, a revision of 2001 classification was proposed and published in 2008 which is followed till date. We observed 344 cases of leukemia i.e. including both acute and chronic leukemia during our study period. Thus, on an average, 76 new cases of leukemia are reported each year. This is significantly higher than the observation made by D’ Costa GG et al. where 242 cases of leukemia were studied over a period of 10 years, thus averaging about 24 new cases of leukemia per year.^8^

Non–Hodgkin lymphoma and leukemia are listed in the top ten leading sites of cancer in many centers in India.^9^ Acute leukemias comprised 28.7% of all leukemias in our study.

This is much lesser compared to D’ Costa et al.’s observations, where they comprised 58%. Thus, there is an obvious geographical variation in the pattern of leukemias within India. The peak age of occurrence of acute leukemia which showed a bimodal pattern compared well with D’ Costa et al.^8^’s study and Anuradha Kusum et al.’s study.^10^

While our study showed only a slight male preponderance (M: F=1.3:1), a higher occurrence in men was noted in D’ Costa et al. and Anuradha Kusum et al.’s studies i.e. 2.7:1 and 2.3:1, respectively.^8^^,^^10^

Our study showed that a definitive subtyping of acute leukemia as AML or ALL, purely by morphology, could be made in only 9 of the cases. We used only 2 cytochemical markers (namely myeloperoxidase and periodic acid Schiff) in the cases wherever cytochemistry was performed. The use of these two cytochemical markers helped in the typing of 8 more cases of acute leukemia. Thus, of the 99 cases of acute leukemia, only 17 cases could be subtyped as AML or ALL on morphology and cytochemistry. 14 of these 17 acute leukemia cases were diagnosed as acute myeloid leukemia because, in all of these cases, the myeloblasts showed diagnostic Auer rods which are crystalline structures seen only in AML or in high grade myelodysplastic syndrome. They are never seen in lymphoblasts.^11^ All the 3 cases of ALL could only be diagnosed after cytochemistry where the PAS reaction showed block positivity. Loffler H, et al. opined that ALL diagnosed by cytochemistry must be confirmed by immunophenotyping in all the cases.^12^ 3 cases during the study period were diagnosed empirically as AML M4/M5. Review of the slides of these cases showed a preponderance of monoblasts and its derivatives with scarce myeloblasts. The monoblasts were large with abundant cytoplasm and an almost round nucleus. Faint granules and vacuolations were seen in some of them. All the 3 cases were negative for cytochemistry with myeloperoxidase and PAS.

In only 40 of the 99 acute leukemia cases, a bone marrow trephine biopsy was done. So, only 40 cases of acute leukemia had trephine biopsies for performing immunohistochemistry. Out of this fourty cases, four cases were excluded from the study population owing to inadequate material for Immunohistochemistry, upon reviewing the slides and tissue blocks. Thus, the final study population included 36 cases.

Immunophenotyping of the leukemic blasts is essential for the diagnosis and confirmation of acute leukemia into one of the two types (AML or ALL). The detection system can either be flow cytometry or immunohistochemistry. Till recently, immune profiling by flow cytometry was the preferred method because it could be performed quickly and allows a precise quantification of the percentage of blasts. The cells can express more than one antigen, which can be identified using different flurochrome labeled antibodies on the same cell.^13^

Paraffin section IHC is a simple tool requiring skilled personnel and very little of equipment. Immunophenotyping of leukemic cells on tissue sections used to be infamous because of lack of consistent results.^14^ However, current IHC systems use multicolor combinations and 3 to 5 different antigens can be identified on the same section. Therefore, WHO in its most recent issue acknowledges that IHC can be used as a diagnostic tool for immunophenotypic analysis.^15^ Literature is chocked with information on various IHC panels used in the subtyping of acute leukemia. However, it is prudent that every laboratory develops its own inventory of IHC panel markers keeping in mind the commonality of types and finances involved.

Anti MPO, CD13 and CD33 are the 3 IHC markers used consistently to detect blasts of myeloid lineage by the 3 major conglomerates in hematology namely European Group for the Immunological Characterization of Leukemias (EGIL), US–Canadian

Consensus Group (USCC) and British Committee for Standards in Hematology (BCSH).^13^ Of these, CD13 and CD33 are best detected by flow cytometry. Anti MPO; however, is best detected by IHC and has a very high specificity for myeloid lineage.^16^ Hence, anti MPO was the first to be included in our panel. We were aware that AML/ M5 may not be picked up. However, AML M5 is rarely reported in our centre and monoblasts are morphologically distinct from myeloblasts.

CD19, CD20 and CD10 are the 3 markers common to all the 3 panels (EGIS, USCC and BCSH) for the detection of lymphoblasts of B cell type. CD19 is commonly used in flow cytometry and is not preferred for IHC studies as reliability tests are incomplete.^17^ CD10 can be detected both by flow cytometry and immunohistochemistry.

However, a proportion of normal hematogones also express CD10.^18^ Hence, CD20 was the 2nd marker to be included into our panel. CD 2 is the only IHC marker that is found in all the 3 panels of the expert groups to detect lymphoblasts of T cell lineage. CD3 and CD7 are common to 2 systems.

Hence, we had to choose between CD2, CD3 and CD7. Chaung SS et al. observed that anti CD3 specifically stained T lymphocytes on paraffin sections.^16^ Hence, CD3 was the 3rd marker to be included in our panel. The last to be included in our panel was TdT. This is a marker of immature hematopoietic and lymphoid cells. It is not lineage specific. It is positive in many of the ALL (B subtype in particular) and a few cases of AML (AML M0, M1).^13^

Prior to the application of IHC on tissue sections, 20 of the 36 cases of the study population could not be typed as myeloid or lymphoid. However, 18 of whom could be clearly categorized as either AML or ALL. Thus, the lineage of 90% of acute leukemias could be identified. This compares well with the observations made by Arber DA et al. who noted that 96% of acute leukemias could be subtyped effectively.

However, their IHC panel included CD34, CD43, CD68, CD79a and HLA–DR in addition to the 4 used by us.^14^ One case expressed both myeloid and lymphoid phenotypes i.e. MPO, CD3 and TdT. The recent WHO classification acknowledges a broad entity called acute leukemias of ambiguous lineage. Acute leukemias which express antigens of more than 1 lineage are also termed as mixed phenotype acute leukemias (MPAL). These are rare and account for 4% of all cases.^19^ The occurrence of MPAL in our study population was 3% which is similar to that in literature.

Results of all the 11 cases of AML and 5 of ALL that were diagnosed by morphology and cytochemistry corroborated well with IHC studies. A much larger correlative study done by Browman GP et al. showed a 99% concordance. The authors opine that even though a morphological diagnosis of the type of acute leukemia is made, IHC has to be done for confirmation as morphological assessment has a high rate of inter observer discordance.^20^

All three cases which were suspected to be AML M4/M5 and were found to be negative for cytochemistry turned out to be AML on IHC. MPO was expressed randomly in about 20–40% of the immature cells. The remainder was negative for MPO and other 3 IHC markers in our study. These could be monoblasts and their derivatives. Antibody against lysozyme may be useful in such cases.^13^

One case was negative for all the IHC markers. 4 types of acute leukemia can be classified for this presentation. They are AML with minimal differentiation, acute monoblastic leukemia, acute megakaryoblastic leukemia and pure erythroid leukemia. Of these, TdT may occasionally be positive in AML with minimal differentiation and acute megakaryoblastic leukemia. We reviewed the morphology and cytochemical results of this case. The blasts showed no evidence of differentiation. They had scant, deeply basophilic cytoplasm and devoid of granules. Prominent nucleoli numbering 1 to 4 were also seen. This morphology clearly ruled out the possibility of AML M5 as monoblasts had a distinct morphology. The absence of irregular or indented nuclear contours, cytoplasmic blebs and pseudopods were against the typical appearance of a megakaryoblast. Cytochemistry showed that the cytoplasm was negative for a PAS reaction; so, pure erythroid leukemia is also unlikely. Hence, it is possible that this is a case of AML with minimal differentiation. Up to 50% of AML cases with minimal differentiation are negative for TdT. Thus, the only way to confirm this is testing CD34, CD38 and HLA–DR because most cases of AML with minimal differentiation are CD34, CD38 and HLA-DR positive.^14^

## CONCLUSION

 Only 9 cases of acute leukemia could be typed as AML or ALL purely by morphology. Another 10 cases could be typed using cytochemistry. The panel used in this study helped to type 90% of acute leukemia cases into AML or ALL and also identify 1 case of AML of ambiguous lineage. Based on our results, we suggest the use of this limited panel of immunohistochemical markers including MPO, CD20, CD3 and TdT for the routine evaluation of all acute leukemias in paraffin embedded tissues. For a resource poor country such as ours, it is easier to type acute leukemia using immunohistochemistry than flow cytometry given the disadvantage of the costs involved with the latter.
